# Tau levels in platelets isolated from Huntington’s disease patients serve as a biomarker of disease severity

**DOI:** 10.1007/s00415-025-12966-9

**Published:** 2025-03-06

**Authors:** Melanie Alpaugh, Juan Lantero-Rodriguez, Andrea L. Benedet, Uriel Manseau, Martine Boutin, Massimo Maiuri, Helena L. Denis, Maria Masnata, Shaline V. Fazal, Sylvain Chouinard, Pedro Rosa-Neto, Roger A. Barker, Kaj Blennow, Henrik Zetterberg, Richard Labib, Francesca Cicchetti

**Affiliations:** 1 Department of Molecular and Cellular Biology, University of Guelph, Guelph, Canada; 2 Institute of Neuroscience and Physiology, Department of Psychiatry and Neurochemistry, The Sahlgrenska Academy at University of Gothenburg, Mölndal, Sweden; 3 Clinical Neurochemistry Laboratory, Sahlgrenska University Hospital, Mölndal, Sweden; 4 Translational Neuroimaging Laboratory, McGill University Research Centre for Studies in Aging, Douglas Research Institute, Le Centre Intégré Universitaire de Santé et de Services Sociaux (CIUSSS) de l’Ouest-de-L’Île-de-Montréal, Montreal, Canada; 5 Department of Mathematical and Industrial Engineering, Polytechnique Montreal, Montreal, Canada; 6 Département de Psychiatrie & Neurosciences, Faculté de Médecine, Université Laval, Quebec, Canada; 7 Department of Clinical Neurosciences, John Van Geest Centre for Brain Repair, University of Cambridge, Cambridge, UK; 8 Department of Movement Disorders, Centre Hospitalier Universitaire de Montréal-Hôtel Dieu, CHUM, Montréal, Canada; 9 Montreal Neurological Institute, Montreal, Canada; 10 Peter O’Donnell Jr. Brain Institute (OBI), University of Texas Southwestern Medical Centre (UTSW), Dallas, USA; 11 Department of Neurodegenerative Disease, UCL Institute of Neurology, Queen Square, London, UK; 12 UK Dementia Research Institute at UCL, London, UK; 13 Hong Kong Center for Neurodegenerative Diseases, Hong Kong, China; 14 Wisconsin Alzheimer’s Disease Research Center, University of Wisconsin School of Medicine and Public Health, University of Wisconsin-Madison, Madison, USA; 15 Centre de Recherche du CHU de Québec - Université Laval, Axe Neurosciences, Québec, Canada

**Keywords:** Blood, Plasma, PBMC, Cognition, Phosphorylated tau, NTA-tau

## Abstract

Tau is a microtubule protein that is known to be hyperphosphorylated and to aggregate in several chronic neurodegenerative disorders. In many cases, in particular in Alzheimer’s disease, the degree of tau pathology has been demonstrated to correlate with cognitive deficits and/or decline. In Huntington’s disease (HD), a dominantly inherited neurodegenerative disorder, both cognitive impairments and abnormal tau expression have been reported to occur, along with the accumulation of the mutant huntingtin protein. In this respect, tau has been shown to be present in the cerebrospinal fluid of individuals with HD and to increase with disease progression. However, how this relates to changes in tau found in the periphery is largely unknown. In this study, we collected blood samples from patients with HD and isolated multiple blood components including plasma, platelets, and peripheral blood mononuclear cells to measure their tau levels and subsequently correlate these to cognitive impairments and disease stage. Our results suggest that the amount of tau, particularly N-terminal tau (NTA-tau) and total tau (t-tau), is elevated in all assayed blood components and that the quantity of tau within platelets, specifically, is strongly correlated with disease severity.

**Supplementary Information:**

The online version contains supplementary material available at 10.1007/s00415-025-12966-9.

## Introduction

Tau is a microtubule-associated protein predominantly expressed in neurons within the central nervous system (CNS) and which frequently accumulates in chronic neurodegenerative diseases [1]. Under physiological conditions, the microtubule-binding and stabilizing functions of tau are tightly regulated by posttranslational modifications. However, in pathological states, tau becomes abnormally phosphorylated and truncated, detaching from microtubules (MTs) and forming intracellular aggregates. Ultimately, the collapse of the MTs and the subsequent disruption of axonal transport result in the loss of cellular integrity and neuronal dysfunction [1]. While such processes are commonly associated with Alzheimer’s disease (AD), there is a large family of disorders, collectively known as tauopathies, in which tau pathology has been described to be an important contributor to disease [1]. In addition, fluid biomarker measurements of tau phosphorylation are an important predictor of clinical severity in AD, even before disease onset. In fact, phosphorylated-tau (p-tau) levels in the plasma are one of the strongest predictive factors of clinical progression to cognitive impairment in the elderly across a 5 year span [2]. Furthermore, t-tau in cerebrospinal fluid (CSF) is associated with cognitive deficits/decline in neurodegenerative disorders other than AD such as Parkinson’s disease (PD) [3] and Creutzfeldt–Jakob disease [4], suggesting that it may be a useful biomarker in a range of disorders, including Huntington’s disease (HD).

HD is a dominantly inherited neurodegenerative disorder resulting from a CAG repeat expansion exceeding 36 in exon 1 of the Huntingtin gene (*HTT*). This mutation leads to the production of a protein with an abnormally expanded polyglutamine tract in the N-terminus which is particularly prone to misfolding. The detrimental effects of the mutant huntingtin protein (mHTT) are well known, but it is not the only misfolded protein thought to contribute to disease. Numerous studies have reported that tau accumulates within highly affected brain regions, such as the striatum and the cortex [5–11], and that tau pathology progresses with increasing disease severity [12–15]. In addition to being present in HD, reduction of tau levels, either through genetic interventions or pharmacological means, have been shown to improve features of disease in two different mouse models, suggesting a role for tau in HD pathology [16, 17]. These results are consistent with clinical studies showing the presence of elevated CSF t-tau levels in patients [15] as well as a relationship between tau levels and cognitive decline and/or brain atrophy in HD [12–14].

While CSF t-tau levels are commonly used as a biomarker in different diseases, recent technological advances have resulted in novel ultra-sensitive immunoassays capable of measuring tau biomarkers in blood [18–22]. In plasma, p-tau 181 and p-tau 231 have demonstrated to be highly specific AD biomarkers associated with amyloid pathology and cognitive decline [18, 23–25]. Additionally, t-tau biomarkers, such as plasma N-terminal tau (NTA-tau), have been shown to increase in AD, showing tight correlations with tau pathology, neurodegeneration, and cognitive decline [21, 26]. While plasma is the most common component of the blood in which tau is evaluated, studies have shown that tau is also present and detectable within blood elements, such as peripheral blood mononuclear cells (PBMC) and platelets [27–30]. In fact, the levels of high-molecular-weight species of tau in platelets have been suggested to have diagnostic sensitivity and specificity of approximately 70% in AD patients, further supporting the utility of peripheral tau as a biomarker for CNS disorders [27].

Very few studies have evaluated peripheral tau in HD, and those that have been performed were conducted in small cohorts or in mice [6, 31]. To determine if peripheral tau may be a useful biomarker of cognitive dysfunction in HD, we collected blood samples and clinical data from HD gene carriers and age-/gender-matched healthy controls (CTRL) at three locations in Canada and in the UK. We herein report on the measurements of tau in multiple blood components and their relationships with various clinical features.

## Materials and methods

### Experimental cohorts

Research ethical board (REB) approval was obtained from local ethics boards at all collection sites (CHU de Québec, #A13-2-1096; CHU de Montréal, #2015-5705; Cambridge Ethics Committee, REC #03/303 and #08/H0306/26; Cambridge University Hospitals Foundation Trust Research and Development Department, R&D #A085170 and #A091246) and all participants provided informed written consent. All subjects also completed a questionnaire about health issues and medication on the day blood was collected. Both HD patients and CTRL were recruited from HD clinics in Quebec City, Montreal and Cambridge. In total, plasma samples were collected from 64 gene carriers and 61 age-matched CTRL (Table 1); however, only a subset of patient samples had sufficiently concentrated PBMC (*n =* 52 HD gene carriers, *n =* 45 CTRL) or platelets (*n =* 33 HD gene carriers, *n =* 29 CTRL) available (Table 1). The HD group included premanifest gene carriers (plasma *n =* 16, PBMC *n =* 9, platelets *n =* 10) and manifest patients (plasma *n =* 48, PBMC *n =* 43, platelets *n =* 23) according to clinical evaluations [5, 40]. Of the manifest patients, a subset was at a more advanced stage of disease (plasma *n =* 17). This population was paired with healthy control samples of similar age and gender to assess plasma tau levels in more advanced disease stages (HD *n =* 11 females, *n =* 6 males, average age = 59.7 ± 11.4; CTRL *n =* 10 females, *n =* 6 males, *n =* 1 unknown, average age = 57.6 years ± 14.7). Comorbidities were identified based on answers provided on the questionnaire, while blood cell counts were obtained from a routine blood work which was concurrently performed for each individual (Table 1) from blood retrieved in dipotassium ethylenediaminetetraacetic acid-coated tubes (BD Vacutainer, Cat#367,861). Any subjects with abnormal blood counts (i.e., blood cell count, hematocrit, mean corpuscular volume, mean corpuscular hemoglobin, and mean platelet volume), as identified by values outside of the reference range [16], were excluded from the study. The mean pathological CAG repeat length within HD patients was 42 ± 2–23. Clinical evaluations, including the total score on the Unified Huntington’s Disease Rating Scale (cUHDRS) [26] and total functional capacity (TFC) [26], were conducted within 6 months of blood collection, with the majority of subjects having this done on the same day as blood sampling (Table 1).

**Table 1:**
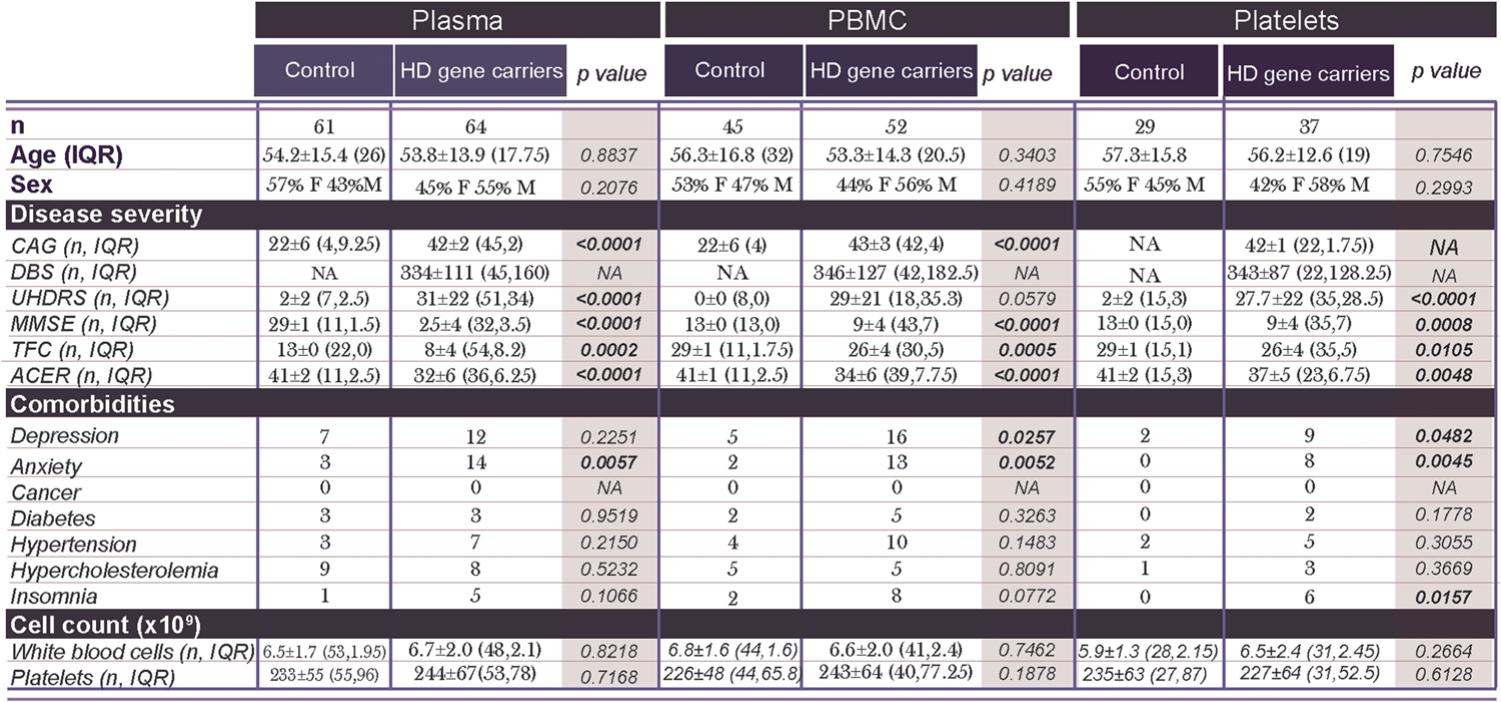
Participant clinical information

Disease severity was evaluated within 6 months of blood sampling. Comorbidities were determined from medical information reported by the participant or caregiver. Statistical analyses: All comparisons between two groups were performed using an unpaired Student’s t test. Comparison of the frequency of males and females within groups was performed using a Chi-square test

*ACE-R* Addenbrookes Cognitive Examination-Revised, *CAG* number of polyglutamine repeats, *cUHDRS* composite Unified Huntington’s Disease Rating Scale, *DBS* Disease burden score, *F* Female, *HD* Huntington’s disease, *IQR* interquartile range, *M* Male, *MMSE* Mini-Mental State Examination, *NA* not applicable/available, *n* number, *PBMC* Peripheral blood mononuclear cells, *TFC* Total Functional Capacity

### Blood sample processing

#### Plasma isolation

All plasma was processed within 2 hours (h) of sampling. For each patient, two citrate blood collection tubes (BD Vacutainer, no. 369714) were centrifuged at 2500 × *g* at room temperature (RT) for 2 × 15 min (min) prior to harvesting the supernatant, aliquoting, and storing at − 80 °C.

#### Platelets

Two citrate-coated tubes (BD Vacutainer, no. 369714) were centrifuged for 10 min at 282 × g. The supernatant was collected and 1/5 of the volume of acid citrate dextrose [ACD, 0.48% (w/v) C_6_H_8_O_7_, 1.32% (w/v) Na_3_C_6_H_5_O_7_, and 1.47% (w/v) C_6_H_12_O_6_)] and 1/50 of the volume of ethylenediamine tetraacetic acid (EDTA, 0.5 M) was added before the complete suspension was centrifuged twice for 2 min at 400 x g and 5 min at 1300 × g. The subsequent platelet pellet was dissolved in 100 µl of Tyrode (7 mM NaHCO_3_, 135 mM NaCl, 3 mM KCl, 0.4 mM NaH_2_PO_4_, 1 mM MgCl_2_, 4.5 mM glucose, and 18.7 mM HEPES buffer) pH 6.5, 900 µl of Tyrode pH 7.4, 200 µl of ACD, and 20 µl of 0.5 M EDTA. The final platelet pellet was centrifuged for 5 min at 1300 × g and dissolved in 100 µl of lysis buffer (Thermo Scientific™, Pierce™ IP Lysis Buffer, no. 87788) with protease and phosphatase inhibitors (Thermo Scientific™, Halt™ Protease and Phosphatase Inhibitor Cocktail 100X, no. 78440). All samples were stored at -80 °C.

##### PBMC

Heparin-coated tubes (BD Vacutainer, no. 367880) were centrifuged at 282 × g for 10 min at RT. Subsequently, the cell pellet was washed in phosphate-buffered saline (PBS) containing 2% fetal bovine serum and isolated using SepMate™ according to the manufacturer’s instructions (StemCell™, no. 15460). PBMC were then homogenized in 200 µL of the same lysis buffer as described for platelets with protease and phosphatase inhibitors and stored at − 80 °C.

### Western blotting

#### Detection of tau in platelets and PBMC

For all immunoblots, positive (prefrontal cortex lysate from mice expressing human tau) and negative controls (prefrontal cortex lysate from tau knock-out mice) were included. For all samples, 40 µg of protein was combined with 1 × 1% (v/v) Laemmli buffer (312.5 mM Tris–HCl, 30% glycerol [Sigma, no. G5516-4L], 12.5% β-mercaptoethanol [Sigma, no. M3148-100ML], 10% SDS, 0.025 M EDTA, and 0.01% Bromophenol blue [Sigma, no. B0126-25G]), and water to a final volume of 40 µL. Prior to loading, all PBMC samples were sonicated in a water bath sonicator for 8 × 5 s and heated at 95 °C for 5 min, while platelet samples were heated at 95 °C for 5 min. Samples were then loaded into 10% SDS polyacrylamide gels and migrated for 90 min at 100 V in running buffer (25 mM Tris HCl, 190 mM glycine [Sigma, no. G7126-5 KG], and 0.1% SDS) and then transferred onto a 0.45 µm polyvinylidene difluoride membrane (GE Healthcare Life Science: 10,600,023) for 1 h at 100 V in transfer buffer (25 mM Tris HCl, 190 mM glycine, and 20% methanol [Fisher Chemical, no. A452-4]). After completion of the transfer, total protein was detected by incubating in ponceau red for 1 min. After the removal of ponceau red by sequential PBS washes, non-specific binding was eliminated by blocking with 3% gelatin extracted from cold water fish skin (Sigma, no. G7041-500G) in PBS for 1 h at RT, followed by overnight incubation at 4 °C with the following primary antibodies: t-tau (1:10,000, Dako, no. A0024) or p-tau 1:5,000 pS199, (Invitrogen, no. 44734G) diluted in 3% fish gelatin in PBS supplemented with 0.1% Tween 20 (PBST) (Fisher Bioreagent, no. BP337-500). The following day, membranes were washed three times for 10 min, incubated for 45 min at RT with IRDye 800CW (LI-COR Biotechnology, no. 926–32,212) and IRDye 680RD (LI-COR Biosciences, no. 926–68,073) antibodies and quantified using Odyssey CLx imaging system (LI-COR Biosciences).

### Simoa

All tau measurements in plasma samples were performed at the Neurochemistry laboratory of the University of Gothenburg (Mölndal, Sweden) using Simoa HD-X instruments (Quanterix). Plasma tau measurements included p-tau 181, p-tau 231, and N-terminal containing tau fragments (NTA-tau). Development and validation of these assays have been described elsewhere [18, 24, 26]. Prior to the assessment of samples, assay beads were suspended in bead diluent, biotinylated detector antibodies in tau 2.0 assay diluent (#101,556, Quanterix) and the enzyme streptavidin-conjugated β-galactosidase (SBG) concentrate (#103,397, Quanterix) in SBG diluent (#100,376, Quanterix). For plasma, randomized samples were thawed, vortexed, centrifuged (4000 g, 10 min), and diluted 1:2 with tau 2.0 assay diluent. An eight-point calibrator curve using recombinant GSK-3β phosphorylated full-length tau-441 (for p-tau 181 and p-tau 231) and non-phosphorylated full-length tau-441 (for NTA-tau) was included in all plates. Two internal quality control (iQC) samples were also present on each plate, before and after the analyzed samples, to control for inter- and intra-assay variability. Repeatability and intermediate precision values in the cohort were < 15%. Calibrators and internal quality control samples were run as duplicates.

### Quantification and analysis

#### Western blots

Quantification of immunoblot band intensity was performed using the Image Lab 6.1 Software (Bio-Rad Laboratories, Inc.) for ponceau signal and the Odyssey Imaging System (Odyssey; Li-Cor, Lincoln, NE) for total tau (t-tau) and p-tau. Protein signal was corrected to ponceau when p-tau and t-tau are shown individually to control for loading discrepancies. For the analysis of p-tau levels, signals were corrected to t-tau. To normalize differences between gels, each independent gel included all experimental groups, and results were calculated as a percentage of CTRL on that gel prior to pooling the results.

### Statistical analysis

Statistical analyses were performed with GraphPad Prism v. 9.0.1 (GraphPad, San Diego, California, USA) or R Studio®1.3.1093. The normality of the data were inspected with the Brown–Forsythe and Bartlett’s test for one-way ANOVA’s as well as the D’Agostino and Pearson normality tests prior to completion of linear regressions. When data were non-normally distributed, non-parametric tests were used where possible, and data were transformed where not possible. Differences between CTRL and HD gene carriers were evaluated using either the Mann–Whitney U test (severe disease comparisons for plasma, and all comparisons for PBMC and platelets) or a one-way ANOVA test with Dunnett’s multiple comparison test (plasma). For all one-way ANOVA and t tests, values that were more than 2 standard deviations away from the mean were identified as outliers and removed from these analyses. In all graphs, bars represent the mean with individual data points indicating biological replicates.

Effects of demographic variables and disease metrics on tau levels were evaluated using simple linear regressions. As tau levels are known to increase with age—and we observed a relationship in our own data (supplementary Table 1)—all data were age corrected using the partial correlation method. For the platelet and PBMC data, a reciprocal transformation was also performed after which the residuals were both randomly and normally distributed. Partial Spearman correlations were performed using the pcor function in R. Outliers were not removed for any of the regression analyses.

## Results

In our data sets, non-disease-associated co-morbidities were equally prevalent in gene carriers and CTRL (Table 1). However, disease-associated variables, such as anxiety, depression, and insomnia, were significantly more prevalent in HD gene carriers, as anticipated [32–34]. While scores pertaining to anxiety and depression were not specifically correlated with tau levels, both are assessed as part of the (cUHDRS) [35]. Potentially confounding variables, such as gender and location of the blood drive (UK vs. Canada), were evaluated with two-way ANOVA models to ensure that they did not affect our data analysis prior to pooling. As no significant differences were observed, these variables were merged in all analyses.

**Fig. 1:**
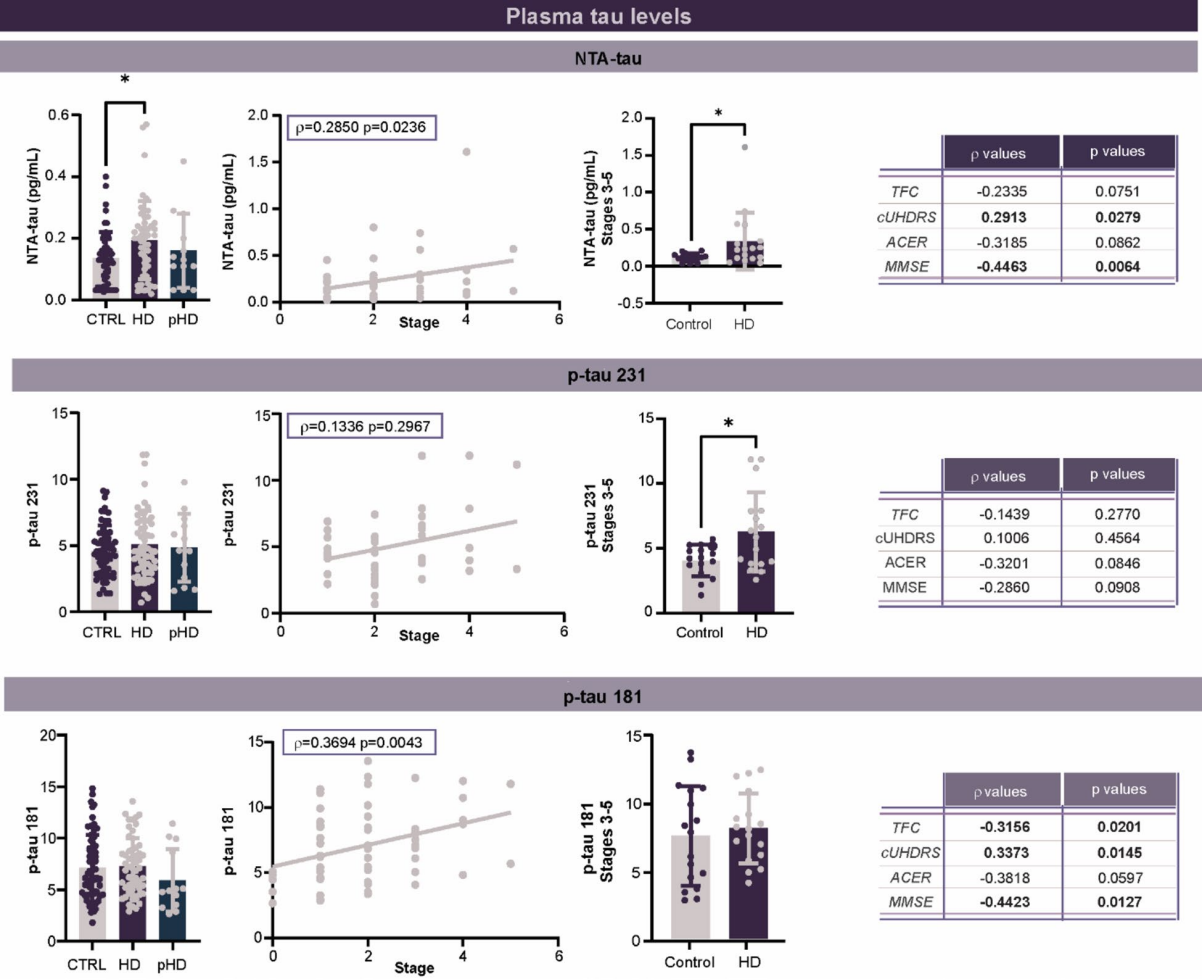
Plasma levels of NTA-tau, p-tau 181, and p-tau 231 increase with disease stage in HD patients. NTA-tau, p-tau 181, and p-tau 231 levels in plasma were all measured by Simoa. Total cohort *n =* 64 CTRL, 55 manifest HD, 13 pHD. Stages 3–5 *n =* 17 CTRL, 17 manifest HD. Statistical analyses were performed using a one-way ANOVA for comparison between CTRL, HD patients and pHD gene carriers, a Spearman test for all correlations and a Student’s unpaired t test for CTRL and stage 3–5 HD patients. * p < 0.05. One way ANOVA results: NTA-Tau between controls, manifest HD and pHD: (F(2,115) = 3.855, *p* = 0.0240); p-tau 231 between CTRL, manifest HD and pHD (F(2,129) = 0.602, *p* = 0.5464); p-tau 181 between controls, manifest HD and pHD (F(2,128) = 1.126, *p* = 0.3274). *CTRL* control, *HD* Huntington’s disease, *MMSE* mini mental state examination, *NTA-tau* N-terminal tau, *pHD* premanifest gene carriers, *p-tau 181* tau phosphorylated at residue 181, *p-tau 231* tau phosphorylated at residue 231, *TFC* total functional capacity

We initially evaluated the levels of NTA-tau and phosphorylated-tau (p-tau181 and p-tau231) in the plasma of HD patients and age/gender-matched CTRL (Fig. 1). For all three forms of tau, premanifest gene carriers displayed similar levels to CTRL but were also not significantly different from manifest gene carriers. In contrast, plasma levels of NTA-tau, but not p-tau 181 or p-tau 231, were found to be increased in manifest HD gene carriers as compared to CTRL (Fig. 1). Previous reports have suggested that tau levels in CSF increase with disease severity [12, 31, 36], so we next explored the possibility of plasma levels also increasing with disease progression by performing a spearman correlation analysis of tau and disease stage after correcting for age. This assessment revealed a significant correlation between NTA-tau and p-tau 181 with disease stage (Fig. 1). Specific evaluation of individuals with advanced disease (Stage 3–5; *n =* 17/group) demonstrated a significant elevation of NTA-tau and p-tau231 when they were compared to age- and gender-matched CTRL.

Having determined that NTA-tau and p-tau levels increase in the plasma of HD patients as disease progresses, we next investigated the relationship between clinical features and tau by performing Spearman correlations between plasma tau measurements and TFC, cUHDRS, the Mini-Mental State Examination score (MMSE), and the Addenbrooke’s cognitive rating scale-revised score (ACE-R) (Fig. 1). These tests were selected as common measures of HD with at least some cognitive component. Specifically, TFC assesses the ability of patients to complete tasks of daily living. The cUHDRS measures four aspects of disease, including motor impairments, cognitive ability, mood, and functional capacity [35]. Finally, MMSE and the ACE-R are general tests of cognition. Significant correlations were observed between plasma NTA-tau and cUHDRS and MMSE, as well as between plasma p-tau181 and TFC, MMSE, and ACE-R. These findings suggest that plasma tau, particularly p-tau181, is related to the degree of clinical impairment observed in HD patients.

**Fig. 2:**
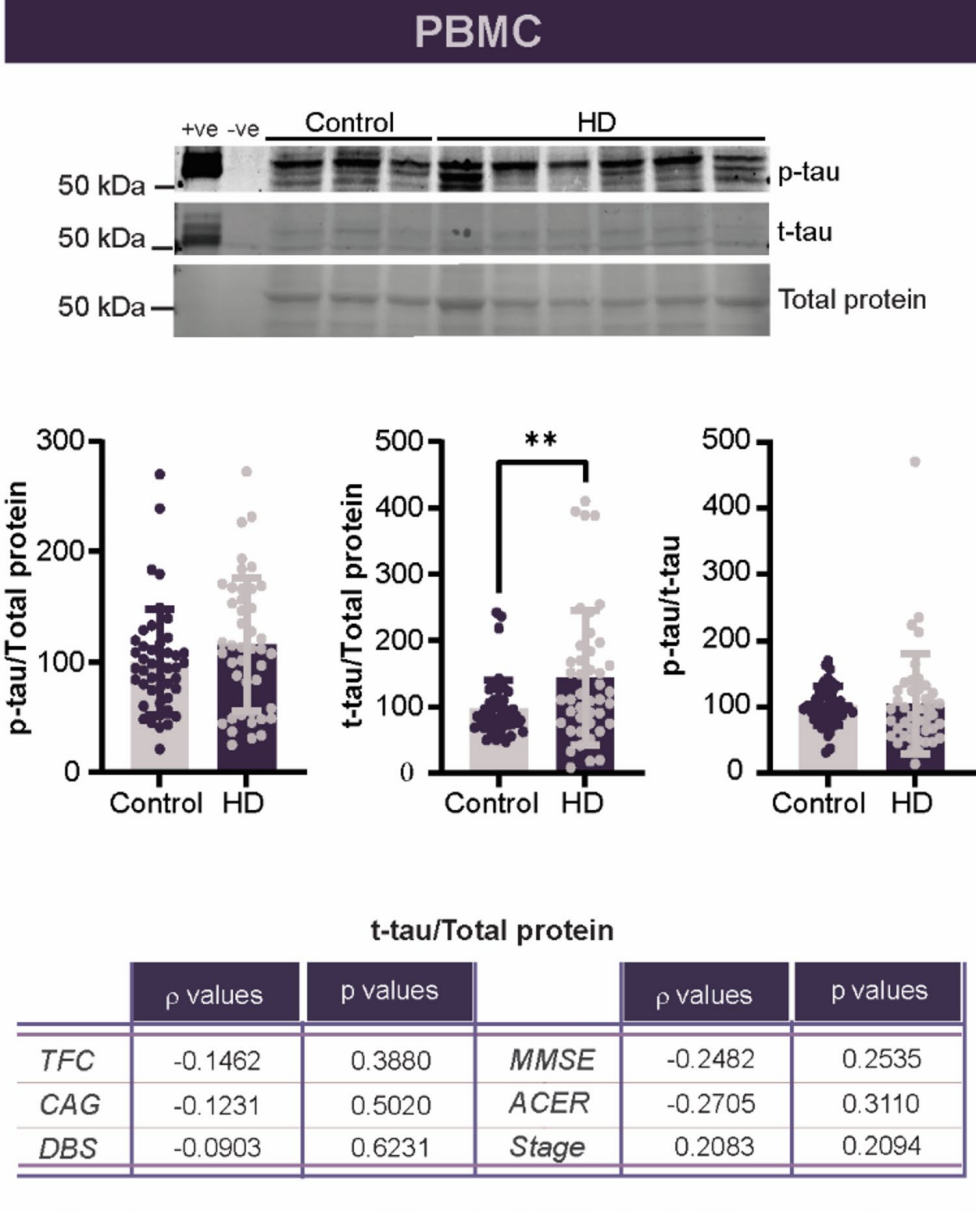
Total tau is increased in PBMC isolated from HD patients. Levels p-tau and total tau were evaluated by western blot. Total cohort *n =* 45 CTRL, 43 HD, 9 pHD. Patients and premanifest gene carriers were pooled in all graphs as no effect of stage was observed. Statistics were performed using a Student’s unpaired t test, or, where variances were unequal, a Kruskal–Wallis test, for comparisons of CTRL to HD gene carriers. Correlations were evaluated using a Spearman test. Spearman’s rho and p values are reported on each graph or in the summary table. * *p* < 0.05. *ACE-R* Addenbrooke’s cognitive exam revised, *cUHDRS* composite unified HD rating scale score, *DBS* disease burden score, *HD* Huntington’s disease, *kDa* kilodalton, *MMSE* Mini-Mental State Examination, *pS199* tau phophorylated at serine residue 199, *TFC* total functional capacity, *WBC* white blood cell. + ve, positive control; -ve, negative control

To continue our evaluation of tau in the blood, we quantified tau within PBMC and platelets as both cell types have been shown to be affected in HD [37–46], in addition to expressing tau. In PBMC, we observed that t-tau, but not p-tau levels, were increased in gene carriers (Fig. 2). Unlike the results obtained with plasma, these changes were not correlated with stage as even premanifest HD gene carriers were characterized by elevated levels (Fig. 2). To understand if other aspects of disease may better correlate with the increase in total tau in PBMC, we looked at levels of tau and disease burden score, CAG repeat length, MMSE, ACE-R, and stage but found no significant relationship between PBMC and any of these clinical measures of the disease.

**Fig. 3:**
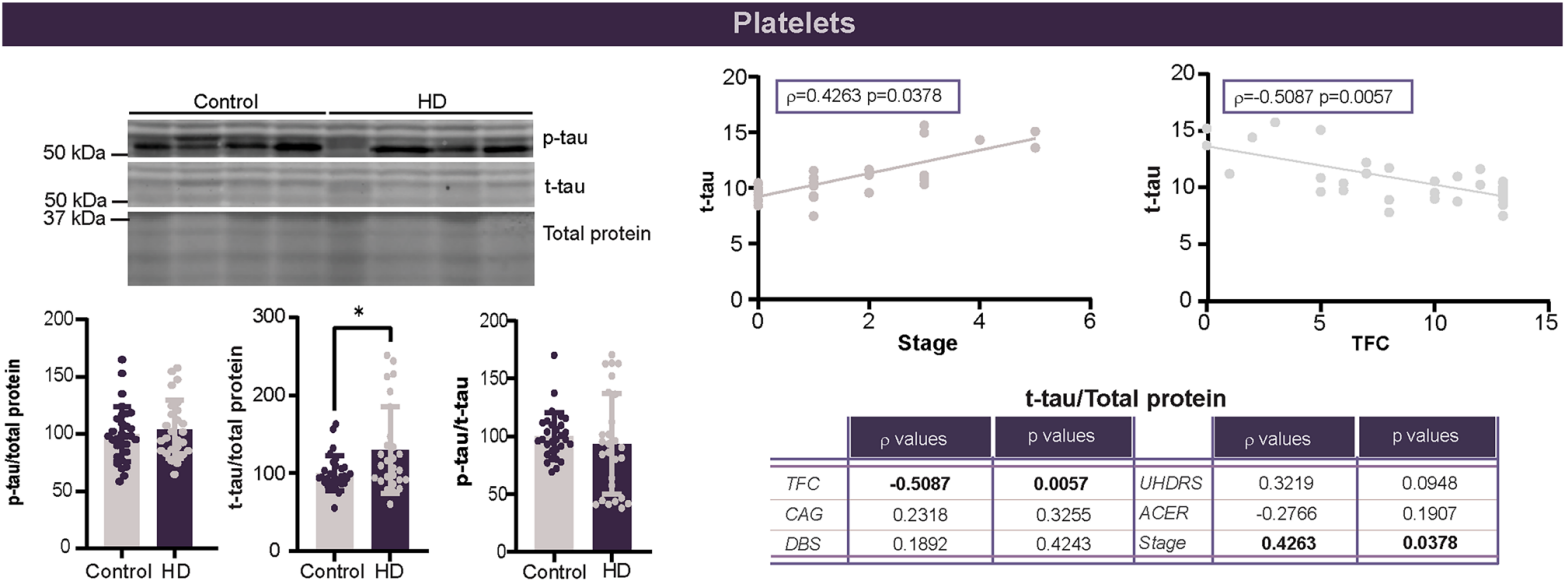
Total tau levels are increased in HD patients and strongly correlate with disease stage and clinical features. Levels of p-tau 199 and t-tau were evaluated by western blot. Total cohort *n =* 29 CTRL, 26 HD, 9 pHD. Graphs below the representative immunoblot include only CTRL and manifest HD patients as a significant correlation between disease stage and t-tau levels was present. Both manifest and premanifest gene carriers are included in all correlation analyses. Levels of t-tau in platelets significantly correlated with disease stage and TFC. Statistics were performed using a Student’s unpaired t test for comparison of CTRL to manifest HD patients. Correlations were evaluated using a Spearman test. * *p* < 0.05. *ACE-R* Addenbrooke’s cognitive examination revised, *cUHDRS* composite unified HD rating scale, *DBS* disease burden score, *HD* Huntington’s disease, *kDa* kilodalton, *MMSE* Mini-Mental State Examination, *pS199* tau phosphorylated at serine residue 199, *TFC* total functional capacity

To complete our analysis of tau in different blood components, we assessed tau levels within platelets. Similar to PBMC, we detected an increase in t-tau in HD platelets but not in p-tau (Fig. 3). Unlike PBMC, this increase was significantly correlated with disease severity (Fig. 3), although a relationship with CAG repeat length and disease burden score was not seen. When TFC, cUHDRS, MMSE, and ACE-R were analyzed, levels of tau within platelets also correlated with TFC (Fig. 3). Combined, these findings suggest that tau levels within platelets may be related to both disease progression and severity.

## Discussion

We assessed NTA-tau, t-tau, and p-tau in different blood compartments from CTRL, premanifest gene carriers, and manifest HD patients. For all three evaluated blood components, a significant increase in t-tau or NTA-tau was observed, which correlated with TFC in platelets and cUHDRS/MMSE in plasma. In plasma, a significant relationship between disease severity and p-tau was also detected. Together, these findings support assessment of blood tau as a potential biomarker of HD severity.

A number of studies have now indicated that tau accumulates as HD advances [12–14] and that reducing abnormal tau can have a beneficial effect on disease course, at least in preclinical models of the disease [16, 17]. However, only two studies have assessed tau levels in HD plasma; one in mouse models [31] and the other in a small clinical cohort [6]. In the study of mouse models of HD, plasma t-tau levels were increased but only in older mice with more severe phenotypes [31]. This is mostly consistent with our findings as there was no increase in premanifest patients, but changes were measurable in the most severely affected group of patients. However, our findings differed from those previously reported in that we observed a significant increase in plasma NTA-tau in all manifest patients and not just in the most severe cohort. This discrepancy could relate to the inherent differences between an animal model and the human disease, although it is also possible that this difference relates to the form of tau measured as we detected NTA-tau and the previous study measured t-tau, which has a weaker relationship with central tau accumulation than NTA-tau [26]. In the one clinical study evaluating p-tau and t-tau, no significant differences were observed between patients and CTRL which contrasts with our findings [6]. The most likely explanation for this divergence relates to the sample size. In our study, we collected plasma from 64 gene carriers and 61 CTRL in contrast to the 10 manifest patients and 10 CTRL which were assayed in [6]. However, it is also possible that differences in age or the specific type of tau evaluated (t-tau vs. NTA-tau, p-tau 181, and p-tau 231) are also responsible for the different results as this previous study only evaluated t-tau [6].

The possibility that the type of tau explains the differences between the two studies is supported by previous work showing that the specific site of tau phosphorylation influences tau physiology and the suitability for tracking different aspects of disease. Specifically, studies of tau physiology have demonstrated that a number of kinases are responsible for the phosphorylation of tau at different residues [47, 48] and that varying the site of phosphorylation can lead to changes in the capacity of tau to bind microtubules and to alterations in cellular localization [49–51]. Similarly, evaluation of tau in patient samples indicates that the specific epitope phosphorylated, or form of tau, changes with disease stage or degree of amyloid pathology. For example, in AD, the most studied forms of p-tau in plasma, p-tau 181, and p-tau 231 [52] both depict variations early in disease course, but p-tau 231 in plasma better separates mild cognitive impairment from AD [53]. NTA-tau also changes early in disease course but has been shown to have a stronger relationship with central tau levels and a weaker relationship with amyloid-beta (Aβ) accumulation than plasma t-tau or p-tau 181/231 [21]. In other neurodegenerative diseases, such as Parkinson’s disease and dementia with Lewy bodies, fewer differences between various phosphorylated forms of tau have been observed with both p-tau 231 and p-tau 181 showing elevations when compared to controls with no neurological disease [54, 55]. These results contrast with our study where a significant increase was only observed in p-tau 231 in patients with advanced disease. However, the presence of significant relationships between p-tau 181 and multiple clinical metrics suggests that both have some association with disease. In our patient group, NTA-tau was the only form of plasma tau that was significantly increased when all manifest patients were compared to controls. This finding could be explained by the strong relationship between NTA-tau and weak relationship with Aβ as Aβ accumulation is not typically associated with HD. To date, insufficient work has been performed in platelets and PBMC to understand how phosphorylation status may relate to disease processes. However, it has been reported that c-terminal tau better discriminates between those with an MMSE score greater than 27 and those with a score between 24 and 27 than p-tau in platelets [29]. This suggests that the exact form of tau evaluated will influence the observed relationship between tau levels and disease severity regardless of the source of tau.

This is further supported by our data set as each form of tau measured has a different relationship with disease. Specifically, NTA-tau correlated with the MMSE and the cUHDRS, platelet t-tau with TFC and disease stage, and p-tau 181 with most clinical measures. For NTA-tau, the greater strength of the relationship with MMSE and the fact that the MMSE is a subcomponent of the cUHDRS suggests that NTA-tau is particularly associated with cognitive impairments in HD patients. This relationship is consistent with reports the NTA-tau correlates well with PET levels of tau [21], and that PET tau levels are linked to cognitive decline [56]. In contrast to NTA-tau, t-tau in platelets was more closely associated with TFC showing no significant correlation with other measures of disease, including more pure cognitive tests. Since reductions in TFC are most strongly connected to age and CAG repeat length [57], this suggests that t-tau in platelets relates to these factors. However, both CAG repeat length and disease burden score, which is calculated using age and CAG repeat length, failed to show significant correlations with t-tau in platelets, which implies that a yet to be identified factor links platelet p-tau and TFC. Finally, p-tau 181 was found to significantly correlate with nearly all clinical measures and the strength of the relationship was similar across the different scales, indicating that it may be more associated with general disease progression than one specific facet of disease.

Of the observed relationships between tau in the blood and various clinical measures, platelet tau levels were the most similar to associations with clinical disease metrics reported for tau in the CSF [14]. While platelets fulfill distinct functions from cells of the CNS, they have often been used as an indicator of nervous system function due to the similarity in the proteomes of these cells and neurons, including the presence of neurotransmitters and their regulatory enzymes [37–41]. In HD, various platelet dysfunctions have been described, including altered uptake or metabolism of monoamines, such as dopamine and serotonin [37–45], altered capacity to clot and to maintain blood-brain barrier integrity [58], changed mitochondrial transport chain subunit expression [59], and activity of nitric oxide synthase (NOS) [60–63]. Of these, the strongest and most consistently reported effect relates to NOS which was shown to associate with age of onset as well as anticipation in age of onset across multiple generations [60–62]. This further suggests that there is a connection between platelet pathology and HD.

While this is the first study to evaluate tau levels in platelets in HD, changes in tau levels in platelets have been reported in other CNS conditions, including AD [27, 28, 64], mild cognitive impairments [29, 65], and depression [28]. In some cases, these alterations correlated with increased behavioral or neurodegenerative features of disease [64, 65] suggesting that tau levels in platelets may be a good indicator of disease stage, including at early stages of disease. Thus, our findings add to previous reports by showing that plasma NTA-tau and platelet tau levels relate to disease stage in HD patients.

## Conclusion

Our study suggests that tau levels increase with disease severity in all evaluated blood compartments in patients with the HD gene. Of the different blood fractions, plasma p-tau 181 and total tau in platelets unveiled the strongest relationship with disease progression and symptom severity. Importantly, the total tau levels in platelets increased steadily across all stages of disease, warranting additional studies to assess the biomarker potential of total platelet tau in HD.

## Supplementary Information

Below is the link to the electronic supplementary material.

Supplementary file1 (DOCX 60 KB)

Supplementary file2 (XLSX 11634 KB)

Supplementary file3 (XLSX 3553 KB)
