# Supplementary material for: Tau levels in platelets isolated from Huntington’s disease patients serve as a biomarker of disease severity
Source: J Neurol. 2025 Mar 6;272(3):254. doi: 10.1007/s00415-025-12966-9 (PMC11885373; doi:10.1007/s00415-025-12966-9)

**Supplementary Table 1. Regression analysis of age.** The influence of age on tau levels was evaluated using Spearman regression analysis


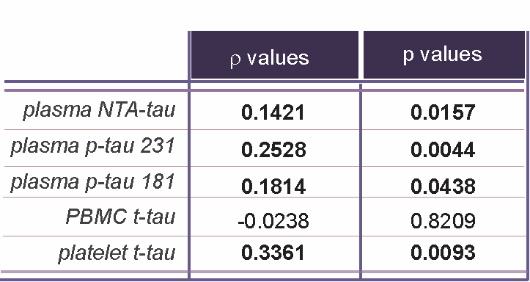

Supplement: Supplementary file 1 — Supplementary file1 (DOCX 60 KB) [file 415_2025_12966_MOESM1_ESM.docx]
